# Virtual simulation of Yue Opera costumes and fashion design based on Yue Opera elements

**DOI:** 10.1186/s40691-022-00300-0

**Published:** 2022-08-25

**Authors:** Kaixuan Liu, Shunmuzi Zhou, Chun Zhu, Zhao Lü

**Affiliations:** 1 School of Fashion and Art Design, Xi’an Polytechnic University, Xi’an, China; 2 College of Fashion and Design, Donghua University, Shanghai, China

**Keywords:** Yue Opera, Opera costumes, 3D restoration of costumes, Virtual costumes, Design

## Abstract

Yue Opera is known as the second most important national opera in China. The costume is an important part of the performance of Yue Opera, which carries the culture and history of Yue Opera. The purpose of this paper is to attempt a virtual simulation of Yue Opera costumes through an understanding and analysis of Yue Opera costumes, as well as to use the extracted elements related to Yue Opera costumes for modern fashion design based on Yue Opera costume style. The research method of this paper is to draw 12 sets of traditional costumes of Yue Opera by understanding and studying the costume culture of Yue Opera and transform them into a 3D digital virtual presentation of the costumes. The costume elements are then extracted for costume design so that the designed fashions can reflect the cultural characteristics of Yue Opera, and then virtual simulation technology is used for costume display to realize the dissemination of Yue Opera costume culture. The use of three-dimensional virtual simulation technology to digitize costumes contributes to the preservation and dissemination of Yue Opera costume culture. Secondly, the design of modern fashion using the concept of Yue Opera plays a role in the preservation and dissemination of Yue Opera costume culture.

## Introduction

Nowadays, the way to observe the traditional costumes of Yue Opera is to search the internet for pictures and images, or to enter a Yue Opera museum and look at the costumes through the windows, but we cannot get close enough to see them and we can only see the costumes in two-dimensional, flat displays. This is inconvenient for the public and is not conducive to the dissemination of Yue Opera costume culture. With the current rapid development of technology, realizing the digital conservation and dissemination of Yue Opera costume culture is an effective way. Text, images and films can be used as carriers to provide documents, materials and stages for the preservation of local opera. Deepening the application and development of science and technology in the cultural field, combining traditional culture with modern technology is a way of inheriting and promoting traditional culture. We can use virtual simulation technology to virtually simulate and restore the traditional costumes of Yue Opera, transforming two-dimensional pictures into three-dimensional virtual costumes, increasing the ways in which Yue Opera costumes can be disseminated and enabling people to observe traditional costumes more easily and in greater detail.

As the main carrier of Chinese cultural heritage, costume contains a rich social and historical cultural connotation. It carries the history and memory of the development of costume, and also reflects the development of traditional costume in different periods from the side. Through the understanding of traditional costumes and virtual simulation restoration, the design of modern fashion using elements such as traditional costume culture is a way to inherit and preserve the culture and history conveyed by traditional costumes. Combining the cultural art of Yue Opera costume with modern fashion has an important impact on the inheritance and development of traditional Chinese opera culture.

The use of 3D virtual simulation of traditional costumes is extremely important and influential in the field of education and the garment industry. In the field of education, 3D virtual simulation of traditional costumes provides a more detailed understanding of the style, structure and pattern of the costumes, and it helps to observe traditional costumes in more detail, promoting traditional costume culture to students. The use of 3D virtual simulation of traditional costumes will enable contemporary young students to gain a deeper understanding of traditional culture and enhance creativity. It will also be easier to bring innovative design ideas to life with the help of 3D virtual simulation software. The extraction and stylization of elements of traditional dress is a source of inspiration for many famous fashion shows. By using traditional dress for 3D virtual simulation, it will be beneficial for the garment industry to obtain information on traditional dress and extract elements for garment design, as well as saving design costs for the garment industry.

Virtual restoration of traditional costumes has been studied by more scholars in this area. Kuzmichev et al. (2018) have restored a Victorian riding skirts using 3D virtual technology. Moskvin et al. (2019) applied 2D and 3D software to recover traditional historical dresses and demonstrated the similarity between the prototypes of historical costumes and their replicas. Moskvin et al. (2020) used a virtual simulation of a Victorian corset as a basis for modelling the body shape of models of the period. Kang et al. (2013) using 3D virtual technology, first modelled a male model and a female model, and then restored two pieces of clothing worn by the upper classes of men and women in the eighteenth century Rococo period. Kočevar et al. (2018) have restored a folk costume of a woman from the Gorenjska region, which includes a hat, costume and shoes. Finally, the virtual costume was evaluated in comparison with the real one and the results were very good.

In this study of virtual simulation of Yue Opera costumes and modern fashion design of Yue Opera elements, the objectives of this paper are: (1) to study Yue Opera and its costumes in a way that restores Yue Opera costumes and Yue Opera-style costume design; (2) to combine Yue Opera costumes with three-dimensional virtual simulation technology to provide another way to preserve and disseminate Yue Opera costumes using modern digital methods; and (3) to analyze and summarize the characteristics of selected Yue Opera costumes, extract traditional elements and expressions such as colors, patterns and shapes from Yue Opera costumes for modern fashion design, combining traditional costume elements with modern fashion to promote traditional opera culture.

## Literature review

### History of Yue Opera costumes and virtual simulation of traditional costumes

Chinese opera is a cultural treasure of China and an art form of the traditional Chinese culture. As one of the five major Chinese operas, Yue Opera originated in Shengzhou, Zhejiang Province, and through its development over the centuries, its stylistic characteristics have become increasingly evident, forming a style that is distinct from other genres. It is this unique style that makes it stand out from other types of operas.

Yue Opera is a local genre of opera from Shengzhou, Zhejiang Province, which has developed through a century of history, during this time it has gone through the following periods (Yu et al., 2018): (1) One day in 1852, Jin Qibing, a peasant, sang a piece in the fields, and others found the singing style simple, smooth and melodious and followed suit; (2) On 27 March 1906, some of the artists set up a simple stage and performed a few plays. This day symbolizes the birth of the small singing class of Yue Opera, the embryonic form of Yue Opera; (3) In 1921, the name "Shaoxing Wenxi" was first published by the newspaper "Xinwen bao". The performance program of this period absorbed the strengths of both Beijing and Shaoxing operas and developed into a large costume opera. As time went on, women also began to enter the theatre, and during this period the terms "Shaoxing male troupe" and "Shaoxing female troupe" emerged; (4) In the 1930s, the term "women's Yue Opera" emerged, which was also the origin of the name. The history of Yue Opera is a history of reform. The reform of Yue Opera was in response to the development of the times and was a spontaneous reform in search of survival; and (5) In 1942 Yue Opera entered a period of reform, led by Yuan Xuefen and others, Yue Opera underwent a more comprehensive reform of its repertoire, mode of composition, costumes and staging. This reform brought Yue Opera into a new phase and had a profound impact on the formation of the style and form of Yue Opera.

After more than 100 years of development, Yue Opera has developed its own unique artistic style, with distinctive costumes that are beautiful, simple and elegant. The art of costume design in Yue Opera can be presented in three ways: (1) The costumes must be based on the content of the play. The character should wear clothes that match his or her identity. Different styles and patterns are used according to the status of the character, and different colors are used according to the psychological activities. In terms of costume realism, it does not over-emphasize historical authenticity and will follow traditional performance habits in designing costumes, but will supplement them with costume patterns and motifs to show their historical authenticity; (2) The costumes are part of the performance of Yue Opera and, as opera costumes, should be designed with the actors' stage movements in mind, as they are the most direct way of conveying emotion; and (3) The costumes of Yue Opera do not over-emphasize historical authenticity, but follow the principle of freehand beauty. The costume design elements of Yue Opera are based on the processing of daily costumes, condensing them and using techniques such as exaggeration and deformation to create an art form that matches the costume. The realistic design of the costumes in terms of shape, color and design adds to the dramatic stage effect.

Costume is an integral component of Yue Opera performance, a product of Yue Opera culture and a bearer of Yue Opera history. The costumes of Yue Opera have experienced the same reforms as those of Yue Opera, and the costumes have gradually become distinctive with the characteristics of beauty, simplicity and elegance. There are many different types of Yue Opera costumes, including ancient costumes, “Zhezi” (gown with a sloping collar), “Pei” (gown with a vertical collar), embroidered robe, skirts and “Kao” (armor), etc., among them, the ancient costumes are the characteristic costumes of Yue Opera. The type of style, color and pattern of the costumes also vary according to the gender, age, status and other factors of the characters of the opera.

The preservation of costumes is not an easy task. In addition to the damage done to them during opera performances, they are also difficult to preserve in their natural state. Most of the fabrics of Yue Opera are mainly silk fabrics, which can’t stand the sun exposure, are vulnerable to moths, and are easy to fade over time. The more time passes, the more difficult to preserve Yue Opera costumes. With the development of science and technology and the rapid advancement of computer technology, it is an effective way to realize the digital conservation of Yue Opera costume culture. As 3D virtual simulation technology is becoming more and more mature, 3D virtual simulation of traditional costumes can not only protect traditional costumes, but also break through the restrictions of time and area to show traditional costumes anytime and anywhere, providing a new method for the promotion of traditional costumes. At the same time its also enriches the scope and value of the use of 3D virtual simulation technology. In a study on the restoration of traditional Chinese costumes by virtual simulation, Jiang et al. (2019) used traditional Chinese costumes as the object of study, firstly to simulate the fabric of traditional Chinese costumes, and then restoring and displaying the costumes using virtual simulation software. Xu et al. (2021) utilized virtual simulation techniques to restore dragon robes from the Qing Dynasty. Yang et al. (2021) used virtual simulation technology to restore the “Xiezhi” round-necked mending costume of the Ming Dynasty by analyzing the structure, fabric, color and pattern of the costume. Song et al. (2021) used the Ming and Qing dynasty Dao robes as an example, and restored the costumes using costume CAD and virtual simulation software. Yuqing et al. (2021) used a portrait of the Song dynasty Queen “Huiyi” as the subject of their study, and used virtual simulation modelling techniques to recover the costume worn by the Empress “Huiyi”. All the studies provide methods for the digitization of traditional Chinese costumes and broaden the avenues for their conservation and dissemination.

### Application of virtual simulation technology

The research on garment virtual simulation is more extensive at home and abroad. With the continuous improvement of 3D virtual simulation technology, it is widely used in various fields, such as interactive design of clothing (Volino et al., 2005; Wang et al., 2021; Xin et al., 2021),interactive virtual real-time fitting of users (Jiang et al., 2019),Evaluation of clothing comfort (Haixia & Yongrong, 2019; Meng et al., 2010),proposal of efficient methods for 3D clothing design (Zhang et al., 2018), improvement of realism of clothing virtual simulation (Cheng et al., 2020; Zhu et al., 2021). Virtual simulation technology application on a 3D virtual costume museum (Lei et al., 2017; Martin et al., 2016; Meier et al., 2021) etc.

The establishment of 3D virtual museums is a boom in various fields due to the combination of technologies such as virtual simulation and 3D modelling. Martin et al. (2016) at The Drexel Digital Museum Project has proposed the viewing of certain costumes through an interactive audience. The viewer can zoom in on each piece and rotate the figure to see the garment from a different perspective. The Italian fashion house Valentino ran an online virtual museum called the Valentino Garavani Virtual Museum in 2011. Meier et al. (2021) created a completely virtual museum of sixteenth century Spanish costumes in 2021, with a 360° view of each garment. 3D virtual Brooklyn Museum, which showcases some of the costumes from the two Netflix shows "The Queen's Gambit" and "The Crown", allows a 360° view of the costumes, click on a costume to see some of the details of the costume in picture form. Lei et al. (2017) used 3D virtual technology to restore and produce Yi costumes and build a virtual costume digital museum to realize the significance of digital conservation of traditional costumes.

The 3D virtual Yue Opera costumes offer great possibilities for the establishment of a 3D virtual Yue Opera museum. At present, the only digital publicity for the Yue Opera Museum is a website for displaying pictures. Using 3D virtual simulation costumes to create a 3D virtual Yue Opera Museum will not only increase the form of digital display of costumes, but also increase interactivity and promote the publicity of Yue Opera culture.

The use of historical costume elements to design modern fashion has become a common practice. Chen (2011) researches Chinese ethnic costume culture and combines the extraction of costume elements with modern fashion design to achieve the inheritance and innovation of ethnic costume culture. Aleksei Moskvin and Evgenii Surzhenko et al. (2017) pointed out that some fashion designs now draw design elements and inspiration from historical costumes. Zhang (2021) analyzed the application of elements of opera costumes in modern fashion design, and designed modern clothes with opera elements, providing research directions and ideas for the application of opera elements in modern fashion in the future. Lee and DeLong proposed the concept of re-birth design (Lee & DeLong, 2018a, 2018b). The concept of re-birth design inspired us for fashion design based on Yue Opera’s elements. Our design based on the costume elements of Yue opera can be regarded as another form of regenerative design.

In the study of virtual simulation of Yue Opera costumes, we found that restoration of opera costumes research has already been conducted (Jin et al., 2021). However, the research in this paper is in the form of restoration of Yue Opera costumes and fashion design of Yue Opera style. Through 3D virtual simulation analysis of Yue Opera costumes, we understand Yue Opera costume culture, extract elements of Yue Opera costume style and carry out fashion design of Yue Opera elements, combining tradition with modernity. This provides an alternative approach to the conservation and dissemination of traditional opera costumes, such as Yue Opera, and also enriches the scope and value of the use of 3D virtual simulation technology.

## Methods

### Technical roadmap

**Fig. 1:**
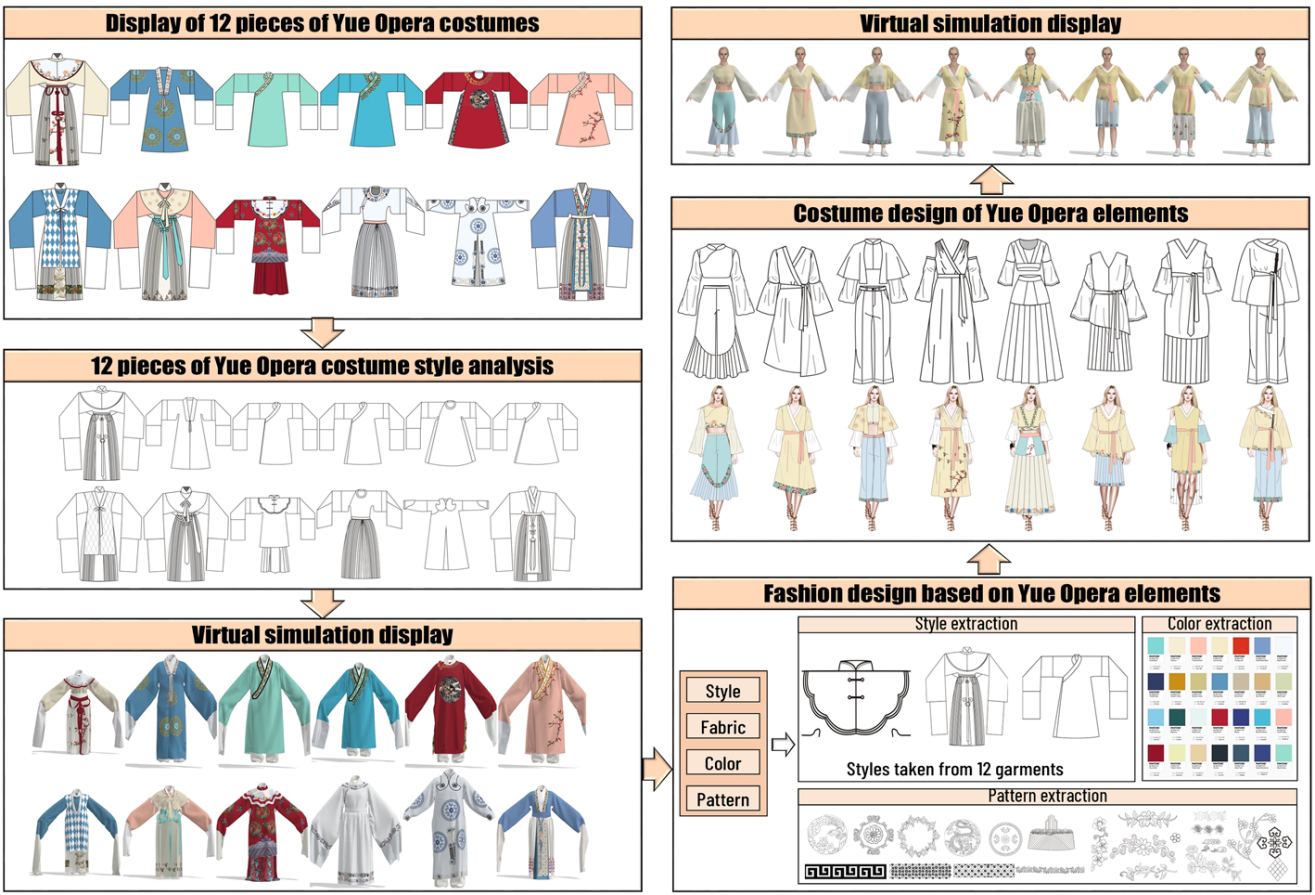
Technical roadmap

Our research is divided into two parts, one is the restoration of the traditional costumes of Yue Opera, and the other is the design and virtual simulation of the costumes of Yue Opera elements. As shown in Fig. 1, the costume styles, fabrics, colors and patterns of the twelve selected Yue Opera costumes were first analyzed to understand the characteristics of Yue Opera costumes. The structural diagram of the style making of Yue Opera was analyzed, and the costume CAD system and virtual simulation technology were used to recover and display the costumes of Yue Opera in 3D simulation.

The second part of the research is shown in Fig. 1. We extracted elements of the style, fabric, color and pattern of Yue Opera costumes by understanding the style of Yue Opera costumes, designed costumes with Yue Opera elements using the extracted elements, and finally used virtual simulation to display them.

### Recovery process

#### Recovery object analysis

**Fig. 2:**
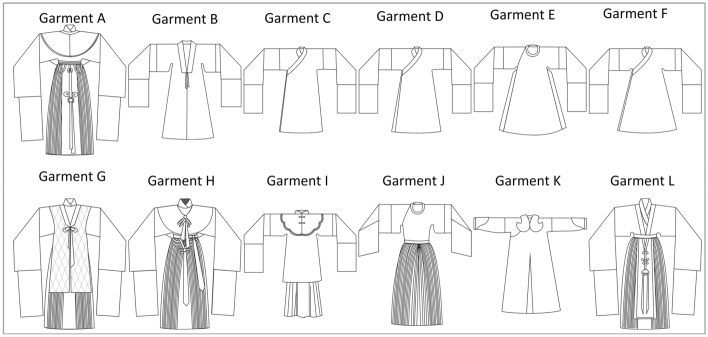
Traditional opera costume style drawing

Costume styles can be analyzed according to the type of costume, as shown in Fig. 2

The ancient costume includes Garment A, Garment G, Garment H and Garment L. They are all X-shaped, and each costume can be divided into a top and a skirt. The upper garment has long sleeves, which are directly attached to the bodice, with water sleeves at the cuffs; a belt and jade pendant are worn at the waist, etc.; the skirts can be divided into pleated skirts and horse-face skirts, which are as long as the feet.

Yue Opera “Zhezi” includes garment C, garment D and garment F. The shapes are H-shaped and A-shaped. The sleeves are attached to the bodice, the collar is slanted, some “Zhezi” have “Kendai” shape under the armpits, the “Zhezi” are long and the bodice has slits on both sides.

Garment B is Yue Opera “Pei”, Its shape is A-shaped, with a centre front placket, a “Kendai” shape under the armpits, water sleeves attached to the cuffs, and slits at the sides of the garment.

Garment E is a modified embroidered robe. The shape is A-shaped, with a round collar, smaller sleeves, sleeves attached to the body piece, with a “Kendai” shape at the junction of the body piece and the sleeves. The robe is slit on both sides and has a hem.

Garment I is a female embroidered robe. The shape is H-shaped, with a cheongsam collar cloud shoulders, with FROG and tassels, the gown has a round collar and small sleeve width, with a “Kendai” shape at the joints, the gown is short and knee-length, and the underskirt is a pleated skirt.

Garment J is a coat skirt. The coat is H-shaped and the skirt is A-shaped. The jacket has a round neckline with a “Kendai” under the armpit and the skirt is pleated. The jacket is generally straight, but to reflect the characteristics of the female “Sheng” character, the straight coat was changed to a coat skirt with a sloping spread, adding to the beauty of the costume.

Garment K is the archery suit. It is A-shaped, with cloud shoulders, a round collar and horse-hoof sleeves. There are slits at the front and back to the waist and slits at the sides of the garment.

#### Color application and pattern composition

Costume colors are visual, giving a strong visual impact and appeal, and can most obviously express the psychological activities of the characters, thus most directly conveying information to the audience and mobilizing their emotions.

**Fig. 3:**
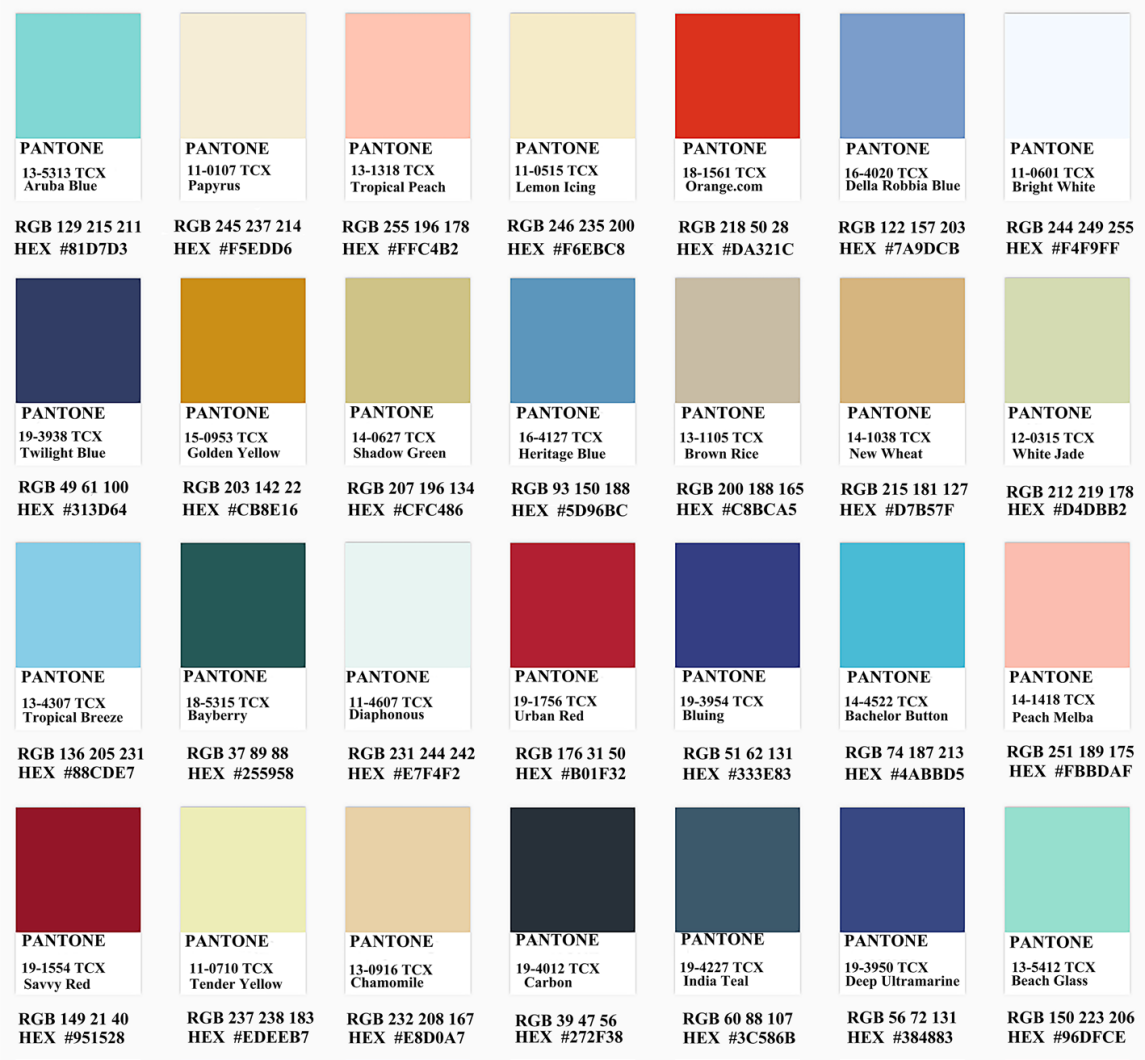
Opera dress color

Traditionally, the colors of opera costumes are divided into "upper five colors" and "lower five colors". The "upper five colors" are "red, black, yellow, white and green"; the "lower five colors" are "blue, purple, pink, lake blue and bronze mist". The color scheme of Yue Opera costumes is no longer limited to the "upper and lower five colors", but has increased the use of many intermediate colors, enhancing the elegance and softness of Yue Opera costumes. The color palette of Yue Opera costumes is rich, as shown in Fig. 3. Among the twelve costumes, the overall color palette is plain and soft, except for embroidered robes and official costumes.

Apart from style and color, Yue Opera costume patterns are another important element of costume. There are many patterns in the costumes of Yue Opera, which not only play the role of decoration, but also symbolize identity and status.

**Fig. 4:**
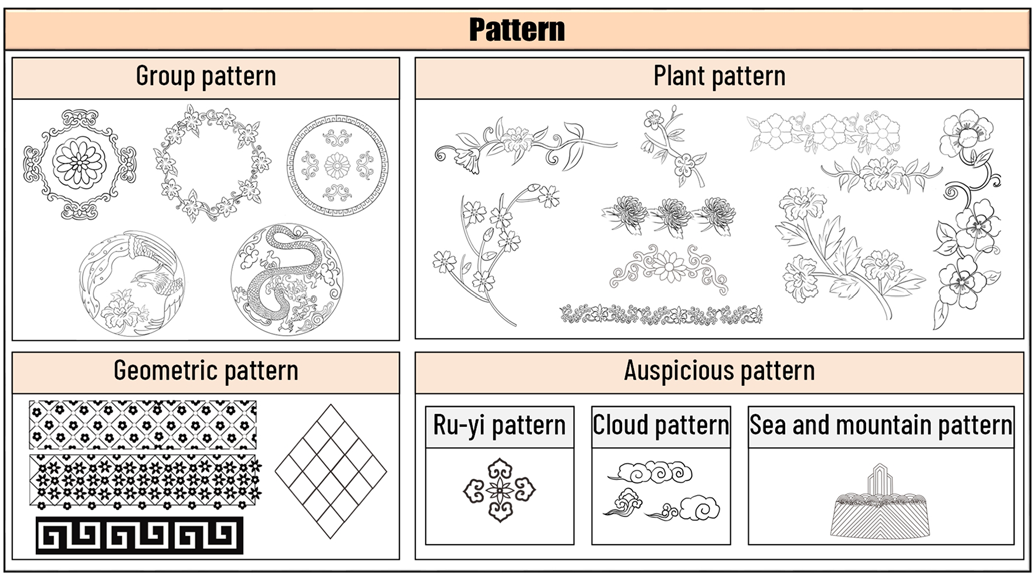
Opera dress pattern

The 12 selected Yue Opera costume patterns are shown in Fig. 4. Classified according to costume style, the ancient costume was mostly decorated with floral patterns, floral and grass patterns, and scroll design reflecting elegance and dignity. The Yue Opera “Pei”, embroidered with posy design, auspicious animal patterns and has a hem embroidered with floral patterns, which symbolizes a noble status. The “Zhezi” worn by the “Wu Sheng” characters are generally embroidered with birds and beasts and posy design, and the “Zhezi” worn by the “Sheng” characters have floral and scroll design, among which the “Four Gentlemen” pattern symbolizes noble and unsullied character. For the male embroidered robe, there are generally patterns such as the round dragon, sit dragon, sea and mountain pattern and cloud pattern to show the majesty and power of the wearer; for the female embroidered robe, the patterns are mainly peony pattern, phoenix pattern, sea and mountain pattern, cloud crane pattern and fret pattern, which are gorgeous and rich; for the modified embroidered robe, the patterns are more simple, with patterns such as fret pattern and grass dragon pattern. If the role is related to religion, there is a lotus flower pattern and other religiously colored motifs. The archery suit is usually embroidered with posy design, floral and grass pattern, etc.

#### Fabric analysis

After the Yue Opera went through reforms, the costumes no longer used the more reflective soft satin, but fabrics of different textures, such as georgette, silk, crepe, etc.

The twelve costumes are mainly composed of silk satin and silk crepe de Chine. The water sleeve fabrics generally require fabrics with a good drape and are easy to whip and whirl, such as silk and koshibo. Underskirt fabrics include: georgette, pure silk, chiffon, koshibo, etc. The fabric of the waistband should have a drape, e.g. silk georgette, etc. Cloud shoulder fabrics include: silk crepe stain plain, silk crepe de chine, silk georgette, etc.

From the above, we can see that the costume fabrics of Yue Opera are mainly silk fabrics, which not only reflects the softness of the costume, but also reflects the style and costume characteristics of Yue Opera.

#### 3D virtual simulation of Yue Opera costumes

**Fig. 5:**
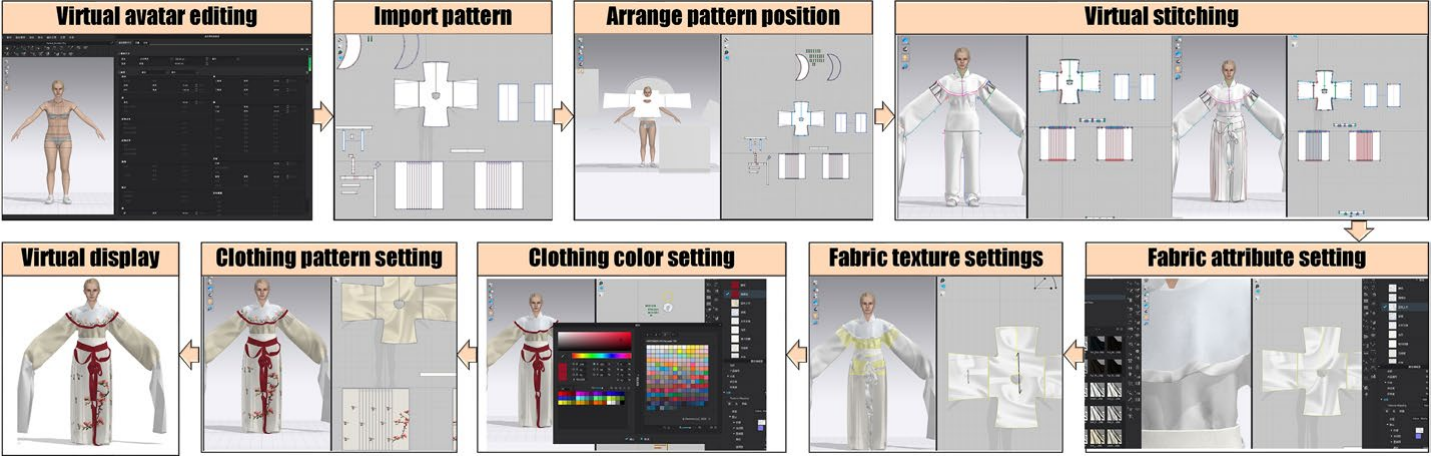
Virtual simulation process display

The establishment of the virtual mannequin data is the basis for costume restoration. As the actors in Yue Opera are generally girls, the mannequin data can be divided into 160 cm and 165 cm as the height data for the “Dan” and “Sheng” characters respectively. Then the Fuyi CAD software is used to draw the garment construction diagram, and the garment pattern is imported into the CLO 3D virtual simulation software through DXF format to fine-tune the garment pattern. The CLO 3D interface can present 2D and 3D versions, allowing you to observe the changes in the 3D interface during the sample adjustment process, making the overall effect very convenient. After adjusting the pattern, the garment was tried on virtually: firstly, arrange the position of the sample; secondly, virtual sewing the clothing pieces in order from inside to outside, and finally carry out the virtual fitting and adjustment of clothing. After the virtual fitting of clothing is completed, the fabric, texture and color of the garment is set after checking that all parts of the garment have achieved a high degree of reproduction. Then fill in the clothing pattern in the virtual simulation software, and finally display the restored clothing, as shown in Fig. 5.

### Design procedure

#### Pre-design

In the pre-design phase, we need to find the source of inspiration for the design. In this paper, we have designed two series of costumes. The designed clothing draws on all the elements of these twelve types of clothing. Using various styles of clothing elements for modern fashion, the designs reflect the results of diversified design. Design 1: The first series of clothes were inspired by the ancient costumes worn by Dan characters and the Zhezi worn by Sheng characters. The clothes are X-shaped and H-shaped, and the patterns are simple botanical patterns and the colors are quietly elegant. Design 2: The second series of clothes were inspired by ancient costumes, Zhezi, archery suit and Pei. The shape of the clothes is A and H, and the pattern includes botanical patterns, auspicious patterns and slightly more complex group patterns, and the colors extracted are simple and elegant.

Then, we should be clear about who the fashion is designed for and for what occasions. Our design is for the daily wear of people who enjoy opera costumes. Due to the fact that, opera costumes are stage performance costumes, they are not suitable for daily wear. Therefore, the costume design in this paper is based on the structure of modern fashionable women's clothing, using extracted elements of opera costumes such as patterns, colors and styles to design the costumes. The use of opera costume culture in modern fashion clothing meets the need for opera costume culture, while being more convenient for daily activities than wearing opera costumes.

The colors of the two series of costumes have been chosen for their simplicity and elegance, in line with the style and culture of Yue Opera. At the same time, the patterns of the costumes are taken from the traditional costumes of Yue Opera, and the placement of the patterns conforms to the arrangement of the traditional costume patterns of Yue Opera, for example, the layout of the side patterns and the layout of the bottom patterns. The style structure of the costumes is also adopted from traditional Yue Opera costumes, such as the cheongsam collar of the female embroidered robe, the pleated sloping collar worn by the Sheng characters, the water sleeves, the lapels of the 'Pei', and the clouded shoulders of the archery suit.

#### Design interpretation

**Fig. 6:**
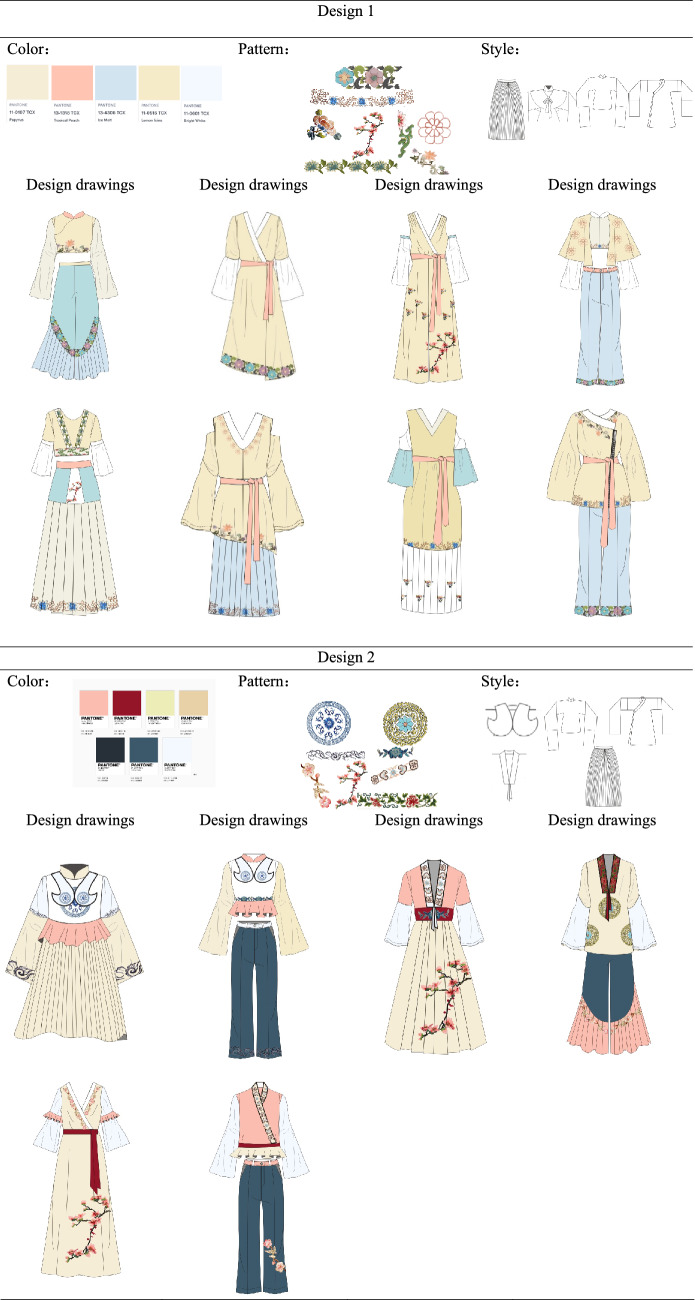
Design source

At this stage, the paper is based on the selected patterns, colors and styles for the design of modern fashion. The colors have been extracted from pantone app and CorelDRAW has been used to draw the clothing style designs and to fill in the colors and apply the patterns. Each designed garment in this paper has been exhibited by the table below, including its inspiration. As shown in Fig. 6.

#### Designing virtual realizations

At the end of the design, we need to make a virtual simulation of the designed garment. In this paper, we draw the structure of 14 garments in Fuyi CAD according to the style of the garment, and then export them in DXF file format and import them into CLO 3D virtual simulation software to complete the virtual simulation of the garment.

## Results

### Virtual simulation of clothing display

This paper analyses the styles, colors, fabrics and patterns of 12 selected traditional costumes of Yue Opera from a costume engineering perspective. After the analysis, style and structure drawings are drawn based on the structural information of the analyzed styles, and then the virtual simulation of the costumes is refined based on the colors and patterns obtained from the analysis.

In the process of analysis, we can learn that the costume fabrics of Yue Opera are mainly silk fabrics, thus reflecting both the softness and suppleness of the costumes and the style and costume characteristics of Yue Opera. The use of intermediate colors in Yue Opera costumes enhances the elegance and softness of the costumes. Most of the patterns used in Yue Opera costumes are floral, botanical and geometric, highlighting the simple, elegant and fresh style of Yue Opera. However, the use of motifs is also related to status, for example, the embroidered robe uses dragon, phoenix and sea and mountain patterns; most of the "Sheng" characters wear "Zhezi" using the "Four Gentlemen: plum, orchid, bamboo and chrysanthemum" patterns, highlighting the high moral character of the wearer. The pattern layout of Yue Opera costumes is also closely related to their simple and elegant style, and in general the pattern layout of Yue Opera costumes will use a border layout, a point layout, and the pattern will not occupy too large an area.

When drawing the structure, the height, bust and waist of each costume is considered according to whether it is a “Dan” or a “Sheng” character, and the length of the arms, width of the cuffs and length of the water sleeves are also designed based on the pictures of Yue Opera costumes on display in the museum. As opera costumes follow the characteristics of "cross shape, integrity and flat body", structural drawings need to be drawn with this in mind. After drawing the structure with the help of CAD and restoring the costume with virtual simulation, the results of the virtual simulation of the Yue Opera costume are presented, with a high degree of visual similarity between the virtual restored costume and the prototype.

**Fig. 7:**
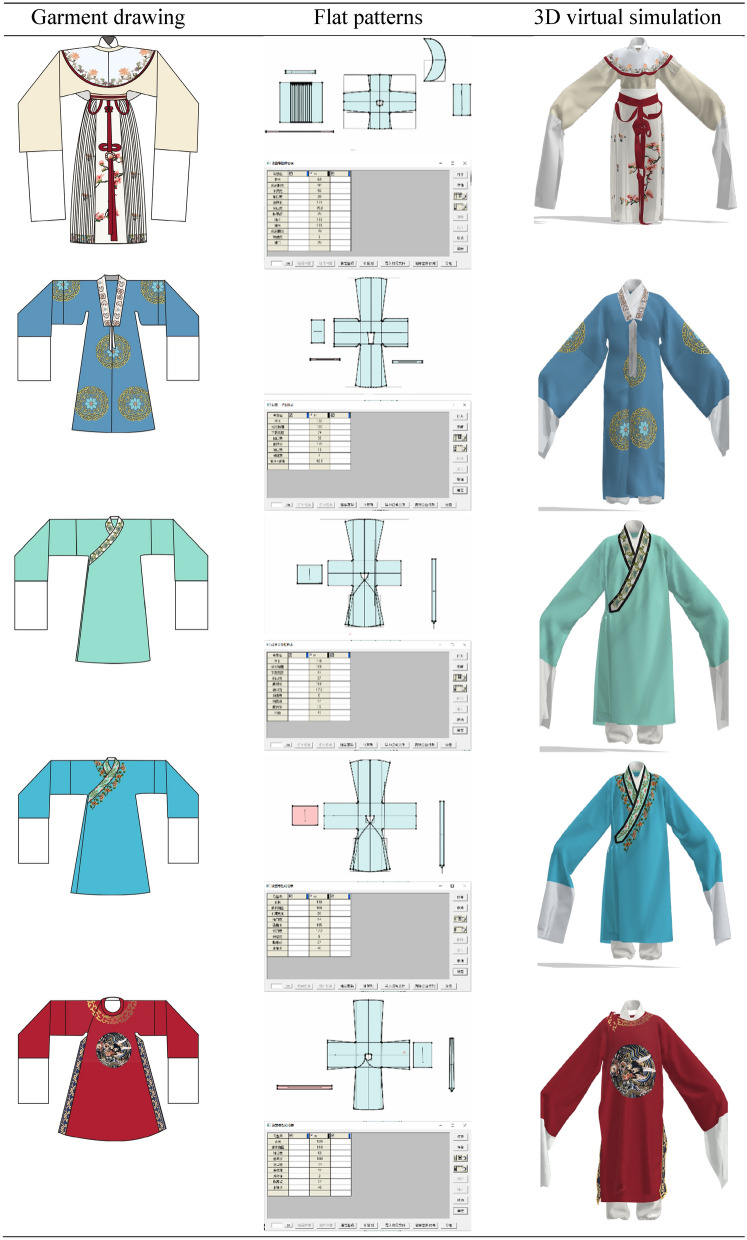

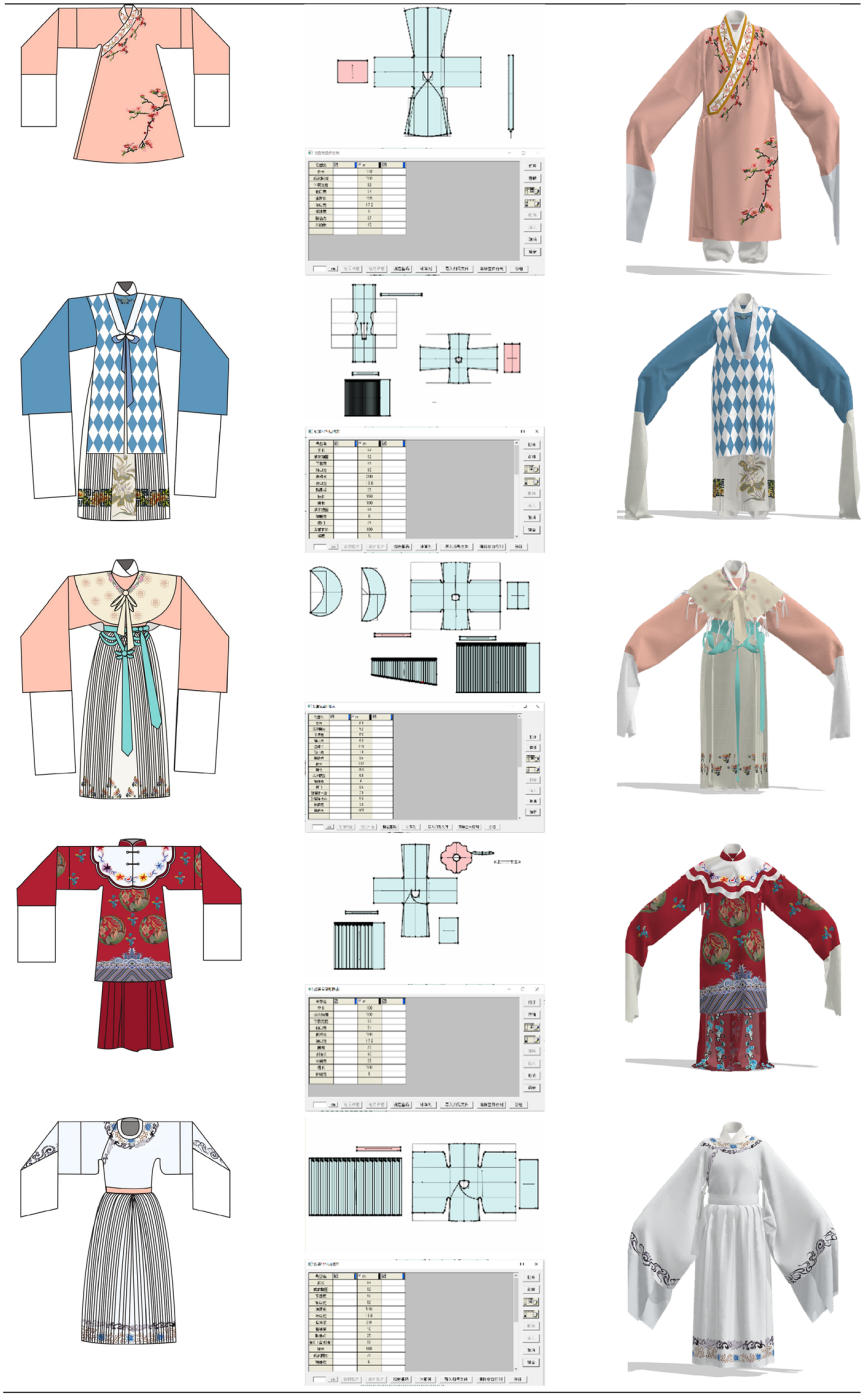

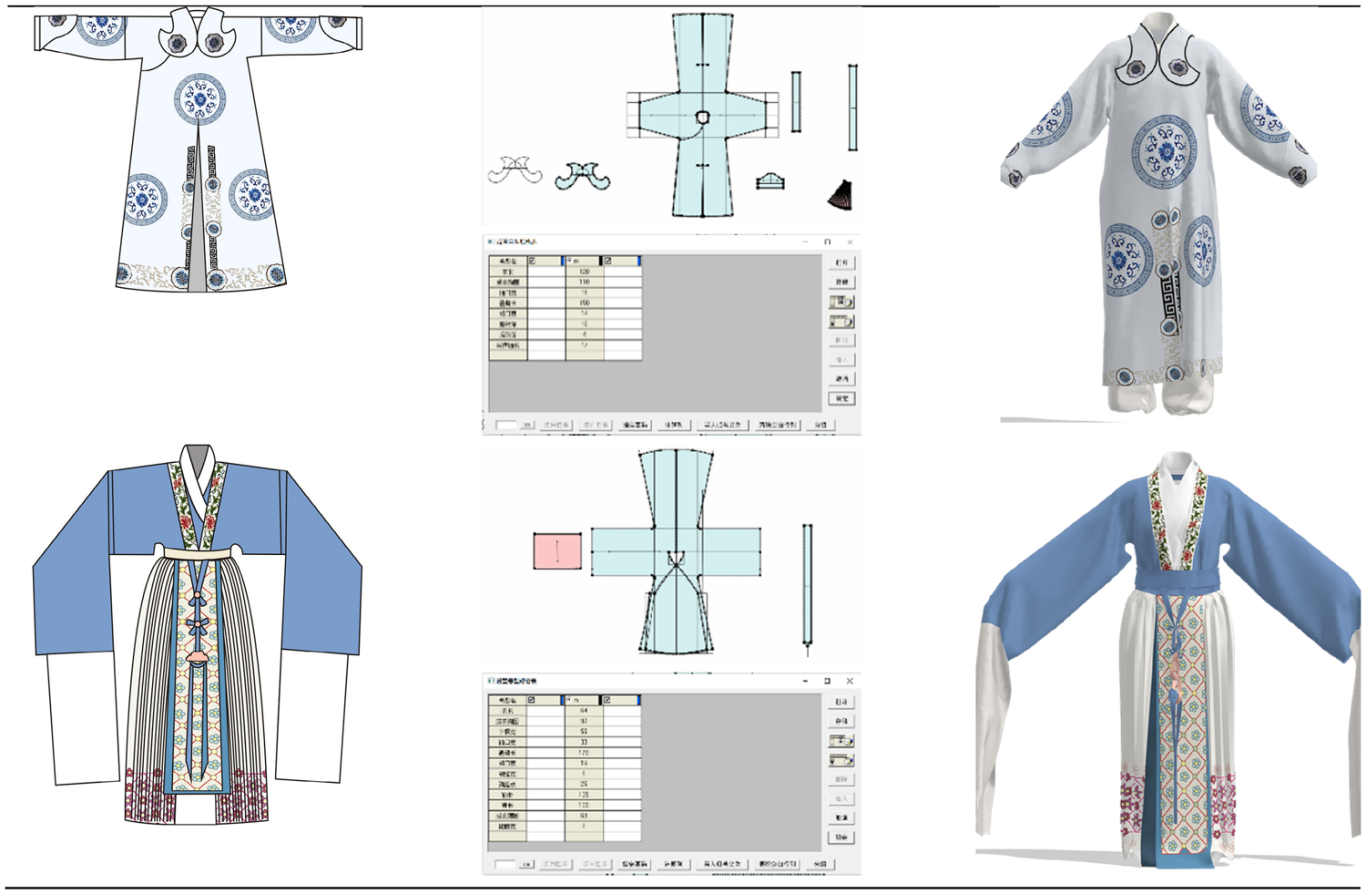
Virtual simulation recovery results

Figure 7 shows the actual garments and the virtual restoration results.

#### Fashion design display

The style, colors and patterns of the designed costumes in both collections have been chosen from traditional Yue Opera costumes. The sleeves are wide, inspired by the water sleeves of opera costumes. The overall shape of the costumes is X-shaped, due to the fact that the shape of the ancient costumes often worn by “Hua Dan” characters are generally X-shaped. The colors of the costume are elegant and soft, reflecting the graceful and poetic nature of Yue Opera and other characteristics. And when setting the position of the flower pattern, the layout of the border pattern commonly used in Yue Opera and other opera costumes is used, and there is no large area of flower pattern. The series of costumes all reflect the simplicity and elegance of Yue Opera costumes.

The traditional costumes of Yue Opera have gone through a history of over a 100 years and have been transformed to form the unique style they have today, and are a treasure in the treasury of traditional Chinese culture. Through the analysis of the traditional costumes of Yue Opera, the spirit contained in the costume culture of Yue Opera is further understood. The design of this series of costumes is based on extracting the traditional costume elements of Yue Opera and incorporating modern fashion style design, blending the traditional opera culture with modern fashion, reflecting the value and artistic charm of the application of Yue Opera costume elements in modern design. It also spreads and protects the culture of Yue Opera costume.

**Fig. 8:**
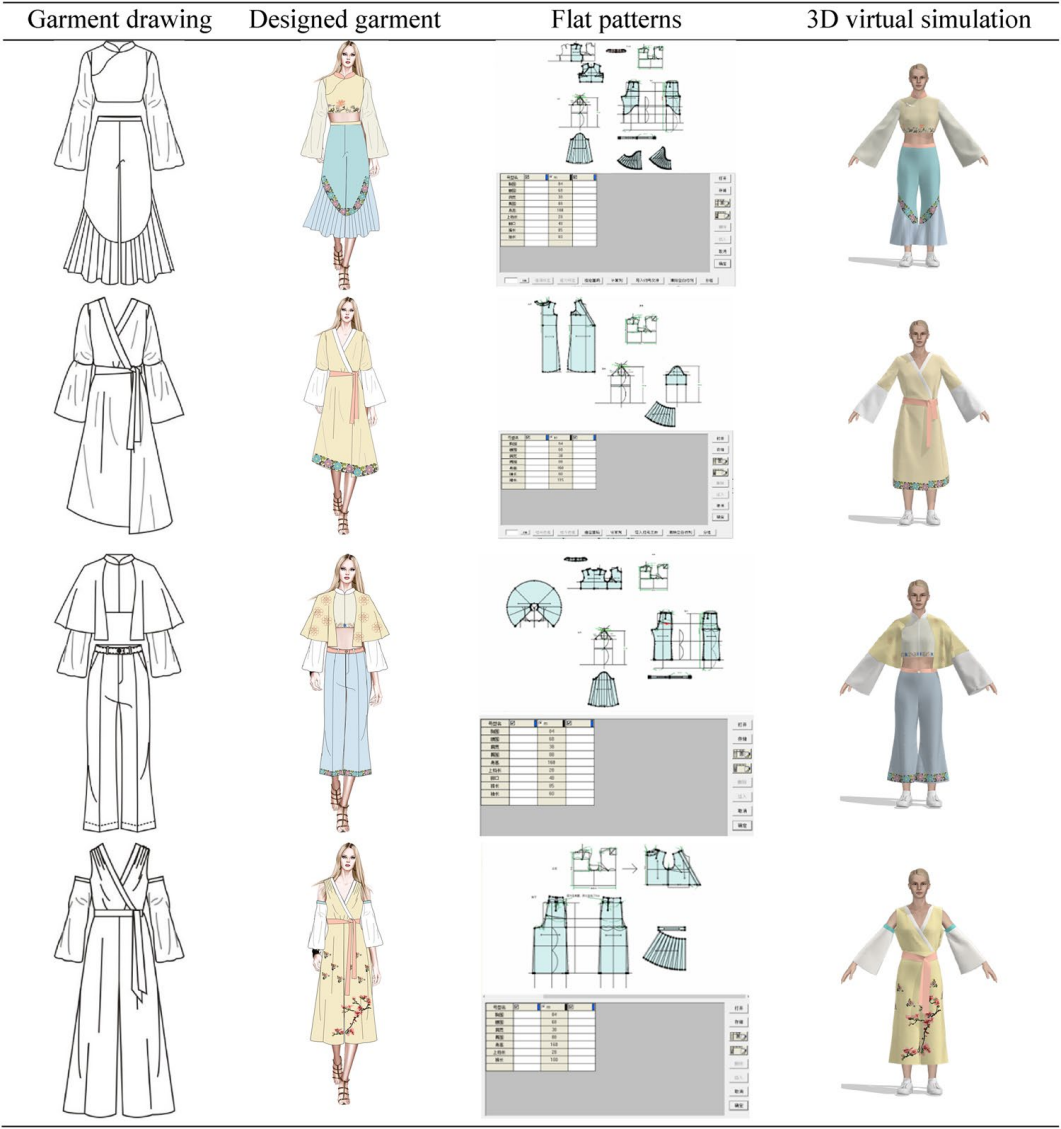

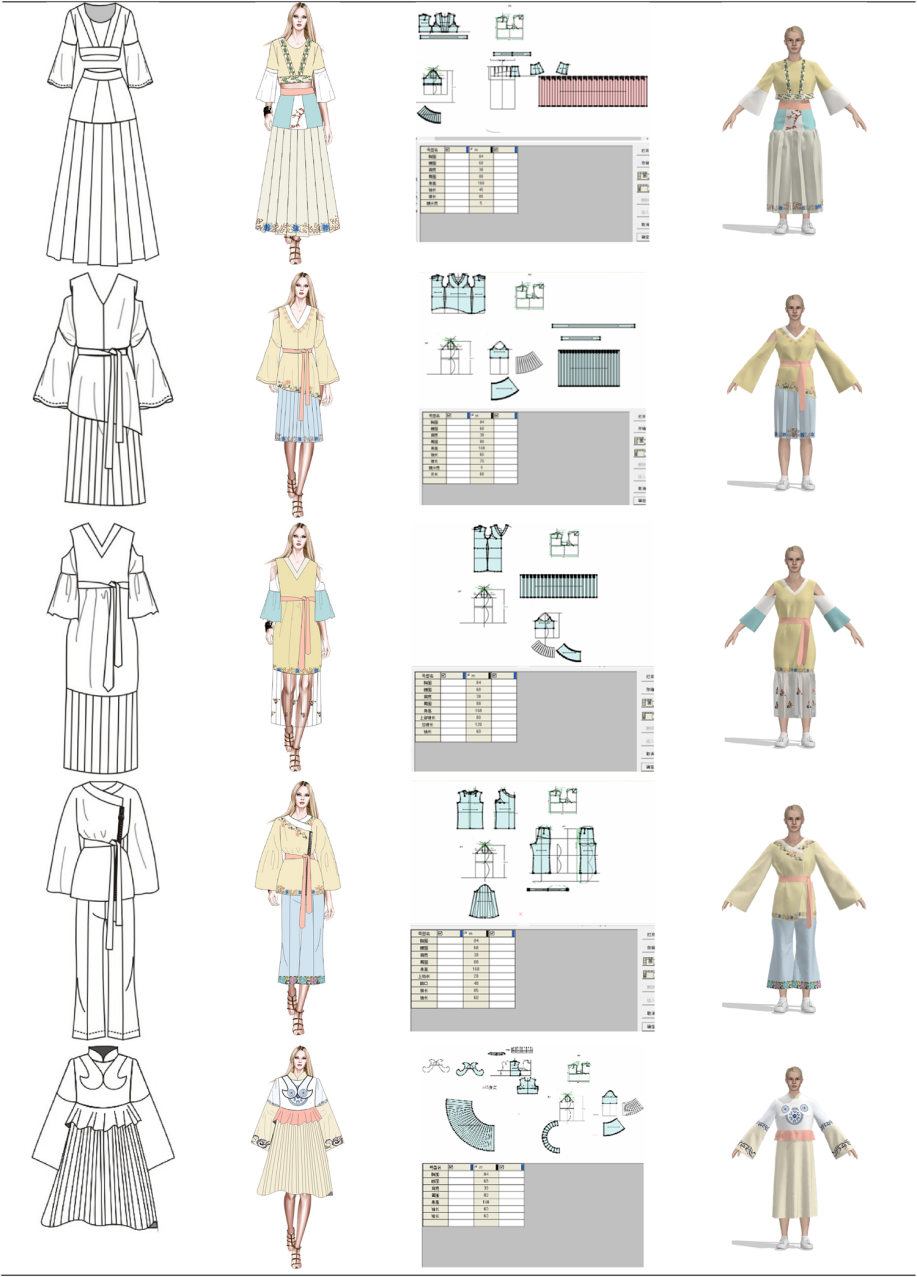

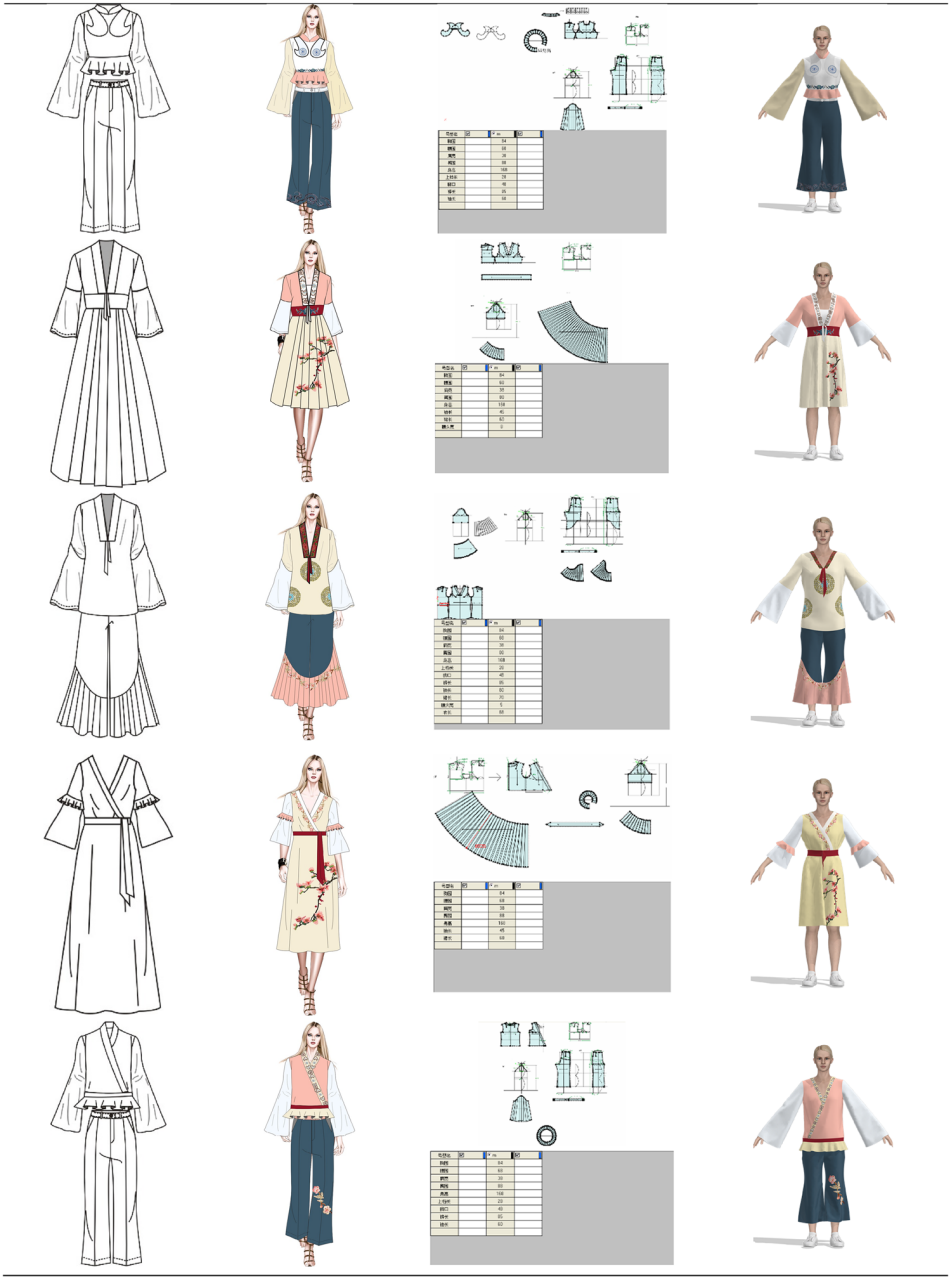
Clothing flat patterns and simulation results

Figure 8 below also shows the design and virtual simulation of costumes with elements of Yue Opera.

## Discussion

The restoration of traditional costume by using 3D clothing virtual display technology has been a hot trend in the field of textile engineering in recent years. Considering current trends, this paper uses virtual simulation technology to virtually restore and innovate the design of Yue Opera costumes. The virtual simulation technology is used to restore the selected 12 sets of Yue Opera costumes, realizing the digital display of Yue Opera costumes and developing the way of preserving and disseminating the culture of opera costumes.

Compared with traditional design methods, three-dimensional virtual costume design has the following advantages: Firstly, the use of three-dimensional virtual technology to design clothes is convenient at any time to use and extract costume elements, can be designed at any time to modify the style, color and pattern of costume, saving design costs. Secondly, 3D virtual costume design can effectively optimize the design process and can display the final design effect statically or dynamically. 3D virtual costume not only saves design time and reduces design difficulty, but also improves the innovation of costume design.

Compared to traditional costume displays, 3D virtual costume simulation has several advantages: firstly, it is of great help to the display and dissemination of traditional costumes of Yue Opera. The virtual simulation of Yue Opera costumes transforms a two-dimensional, flat display into a three-dimensional, 360° virtual display. People interested in traditional Yue Opera costumes can quickly access costume information anytime and anywhere via the internet, which not only breaks through the limits of distance and saves time costs, but also allows people to observe the local patterns of Yue Opera costumes in greater detail. Secondly, it effectively promotes the development of Yue Opera costumes. For the Yue Opera costumes, this paper provides an alternative way of digitally exhibiting the costumes, allowing the culture of Yue Opera costumes to be preserved and disseminated with the help of computer technology. Although museums have protected the costumes as much as possible when exhibiting them, they are not immune to some natural damage. Finally, it contributes to the establishment of a digital 3D virtual Yue Opera museum in the future. This technology can be used by Yue Opera museums to display digital Yue Opera costumes in museums, not just staying with the static display of general museum collections, increasing interactivity, effectively spreading Yue Opera culture and reducing the cost of costume displays.

The virtual simulation of Yue Opera costumes is only one part of the costume virtual simulation research, and the digital representation of Yue Opera costumes provides a technical means of displaying traditional Yue Opera costumes. Moreover, 3D virtual fashion design is bound to be a trend of fashion design in the future. The whole research process of this paper is completed by computer. The simulation method and innovative design methods of Yue Opera clothing can be applied to other clothing styles. The interactive virtual museum and dynamic virtual Yue Opera performance can be constructed by combining the 3D Yue Opera costume’s models with Virtual Reality (VR), Mixed Reality (MR) and Augmented Reality (AR). Through the application of 3D engines such as Unity 3D and UE5, the program of Yue opera performance and virtual museum can be developed. Audiences can watch opera performance at home through VR/MR/AR glasses. Students majoring in clothing can also study the style, color and pattern of Yue Opera clothing through virtual museum. Today, COVID-19 is sweeping through the whole world, this method provides more choices for people who study and live at home. Yue Opera is an important part of Chinese traditional costume culture. Through the innovative design of Yue Opera clothing, we can not only inherit and carry forward Chinese excellent traditional culture, but also provide more design materials for fashion design. Due to space constraints, we will continue to further study along the above directions in the future.

## Conclusions

In this paper, the traditional costumes of 12 pieces of Yue Opera were selected for virtual restoration. At the same time, through the understanding of the history and culture of Yue Opera and costumes, elements are extracted from the restored costumes, and fashion design of Yue Opera costume elements and virtual simulation are carried out.

The significance of this paper, in terms of theoretical value, is that at present there is not much research about restoration of Yue Opera costumes and costume design of Yue Opera elements. This paper studies Yue Opera costumes by restoring them and designing Yue Opera-style costumes, providing another way to disseminate and protect Yue Opera costumes in China, as well as providing material for modern fashion design. In terms of cultural value, costume is an integral component of Yue Opera performance and a product of Yue Opera culture, and the virtual restoration of Yue Opera costumes is another way of passing on Yue Opera culture. Secondly, applying elements of Yue Opera costume to modern costumes not only promotes Yue Opera culture, but also highlights the cultural connotations of traditional opera costumes. In terms of practical application value, the continuation and promotion of opera culture cannot be achieved without the support of technology. The way of preserving opera costumes is more complicated and difficult. This paper uses virtual simulation technology to restore the costumes of Yue Opera, which helps the development of a virtual museum of Yue Opera, and enriches the scope and value of the use of three-dimensional virtual simulation technology. At the same time, combining elements of Yue Opera costumes with modern fashion, the traditional opera costume culture is promoted and preserved in a new way. Secondly, after understanding and learning about the costumes of Yue Opera, designers can draw inspiration from the elements of opera costumes to enrich their own designs.

In this paper, we only carry out static simulation restoration of Yue Opera costumes. Only dynamic simulation can more intuitively express the culture connotation of Yue Opera costumes. Therefore, the dynamic virtual simulation of Yue opera costumes will be an important research direction in the future. Moreover, the fashion design method based on Yue Opera clothing’s elements is still the traditional design method. There is no innovation in the design methodology. Currently, artificial intelligence aided design is an important design method. Therefore, we can combine artificial intelligence algorithms to carry out intelligent design based on Yue Opera clothing’s elements in the future.

## Data Availability

The data (patterns, flats, 3D restored clothing, etc.) used and analyzed during the current study are available from the corresponding author on reasonable request.

## Declarations

### Author information

Kaixuan Liu is a Professor at the School of Fashion and Art Design, Xi’an Polytechnic University, Xi’an, 710048, China. Shunmuzi Zhou is a Graduate student at the School of Fashion and Art Design, Xi’an Polytechnic University, 710048, China. Chun Zhu is an Assistant Engineer at the School of Fashion and Art Design, Xi’an Polytechnic University, 710048, China. Chun Zhu is also a PhD Candidate at the College of fashion and design, Donghua University, 200051, China. Zhao Lü is a Professor at the School of Fashion and Art Design, Xi’an Polytechnic University, 710048, China.
